# Expression Pattern and Subcellular Localization of the Ovate Protein Family in Rice

**DOI:** 10.1371/journal.pone.0118966

**Published:** 2015-03-11

**Authors:** Hui Yu, Wenzhu Jiang, Qing Liu, Hui Zhang, Mingxin Piao, Zhengdao Chen, Mingdi Bian

**Affiliations:** Laboratory of Soil and Plant Molecular Genetics, College of Plant Science, Jilin University, Changchun, 130062, China; University of Antwerp, BELGIUM

## Abstract

The *Arabidopsis* ovate family proteins (AtOFPs) have been shown to function as transcriptional repressors and regulate multiple aspects of plant growth and development. There are 31 genes that encode the full-length OVATE-domain containing proteins in the rice genome. In this study, the gene structure analysis revealed that *OsOFP*s are intron poor. Phylogenetic analysis suggested that OVATE proteins from rice, Arabidopsis and tomato can be divided into 4 groups (I–IV). Real-time quantitative polymerase chain reaction (RT-qPCR) analysis identified *OsOFP*s with different tissue-specific expression patterns at all stages of development in the rice plant. Interestingly, nearly half of the total number of *OsOFP* family was more highly expressed during the seed developmental stage. In addition, seed developmental *cis*-elements were found in the promoter region of the *OsOFP*s. Subcellular localization analysis revealed that YFP-OsOFP fusion proteins predominantly localized in the nucleus. Our results suggest that OsOFPs may act as regulatory proteins and play pivotal roles in the growth and development of rice.

## Introduction

Development of an organism is based on the temporal and spatial regulation of gene expression, in which transcription factors act as switches of the regulatory cascades [1]. Transcription factor genes comprise a substantial fraction of all eukaryotic genomes [2]. In the *Arabidopsis thaliana* genome, there are 1922 known and predicted transcription factors, representing >5% of the total number of genes for this genus, distributed across 64 families [2,3]. It has been reported that members of many large transcription factor families play essential roles in plant development [4–7]. The highest representative in plants is the MADS-box transcription factor family. Members of this family have multiple functions in the transition to flowering, floral organ identity, gametophyte and seed development, fruit development, and lateral root formation [8]. Recently the *Arabidopsis* ovate family proteins (AtOFPs), forming a plant-specific transcription factor family, were found to control multiple aspects of plant growth and development [9–12]. There are 18 genes in the *Arabidopsis* genome that encode proteins with a predicted OVATE domain. The *OVATE* gene was originally cloned from the tomato plant in which a single mutation led to a premature stop codon in this gene, thus causing the transition of the tomato fruit from round- to pear-shaped [13]. In addition, overexpression of the ovate gene from the wild-type round-fruited line in pear-shaped fruit producing line conferred unevenly reduced size of floral organs and leaflets [13]. The tomato OVATE protein contains a C-terminal domain of approximately 70 amino acids that are conserved in tomato, *Arabidopsis*, and rice plants [13]. This domain was designated as the OVATE domain, and the proteins containing this conserved domain were designated as ovate family proteins (OFPs), which can be found exclusively in plant proteins [12].

Hackbusch et al. (2005) reported, for the first time, that 9 members of AtOFPs interact with 3-aa loop extension (TALE) homeodomain proteins, fundamental regulators of plant meristem function and leaf development, and can be cofactors in regulating the subcellular localization of TALE proteins [9]. A systematic function analysis of AtOFPs has been done since 2007 [10–12,14]. It was reported that AtOFP1 plays a key role in suppressing cell elongation by acting as an active transcriptional repressor. The *Arabidopsis* overexpressing *AtOFP1* exhibited a phenotype of reduced length in all aerial organs, including the hypocotyl, rosette leaf, cauline leaf, inflorescence stem, floral organs, and silique. The expression of the gibberellin biosynthesis key enzyme gene *AtGA20OX1* was suppressed in *AtOFP1* overexpressed *Arabidopsis* plants [9,11]. Other *AtOFP*s overexpressed also confer abnormal morphological phenotypes [12]. The Arabidopsis OFP4 plays a role in regulating secondary cell wall formation through its interaction with KNOTTED1-LIKE HOMEODOMAIN PROTEIN7 (KNAT7) [12]. AtOFP5 has been shown to negatively regulate the activity of a BLH1-KNAT3 complex during early embryo sac development [14]. There are 31 *OFP* genes (*SlOFP*s) predicted in tomato genome [15]. The expression patterns of 17 *SlOFP*s were examined tissue-specifically in wild tomato. *SlOFP07*, *SlOFP08*, *SlOFP14*, *SlOFP18*, *SlOFP20*, *SlOFP29* and *SlOFP31* were higher expressed in the reproductive organs than those in the vegetative organs. *SlOFP10*, *SlOFP13* and *SlOFP22* were specifically expressed in the seedling stage such as in root, hypocotyl and young leaf [15]. These imply that *SlOFP*s have a specialized function in plant development [15].

As *OFP*s are proposed to encode a novel transcription factor class and regulate plant development broadly, further genetic and biochemical studies of the function of these genes in the important food plants are needed to provide a better comprehension for their roles in plant evolution and domestication. In general, transcription factors (TFs) are expected to be excellent candidates for modifying complex traits in crops because they naturally act as master regulators of cellular processes [16]. The functional study of plant transcription factors has become increasingly popular in recent years. Rice (*Oryza sativa*) is one of the most important food crops worldwide and a model for genomic research in cereals. Some transcription factors have been identified to play prominent roles in many developmental processes in rice [17–19]. However, little is known about the molecular characteristics of OVATE-containing proteins in rice. The results of this study display the bioinformatic characteristics of ovate family proteins in rice (OsOFPs), tissue-specific expression profile, subcellular localization and hormone-responsive expression pattern of each member in this family. Our data provide a very useful reference for the functional analysis of the OFP family in rice.

## Results

### Chromosomal Distribution of *OsOFP*s and Evolution of *OFP*s in Plants

**Fig 1:**
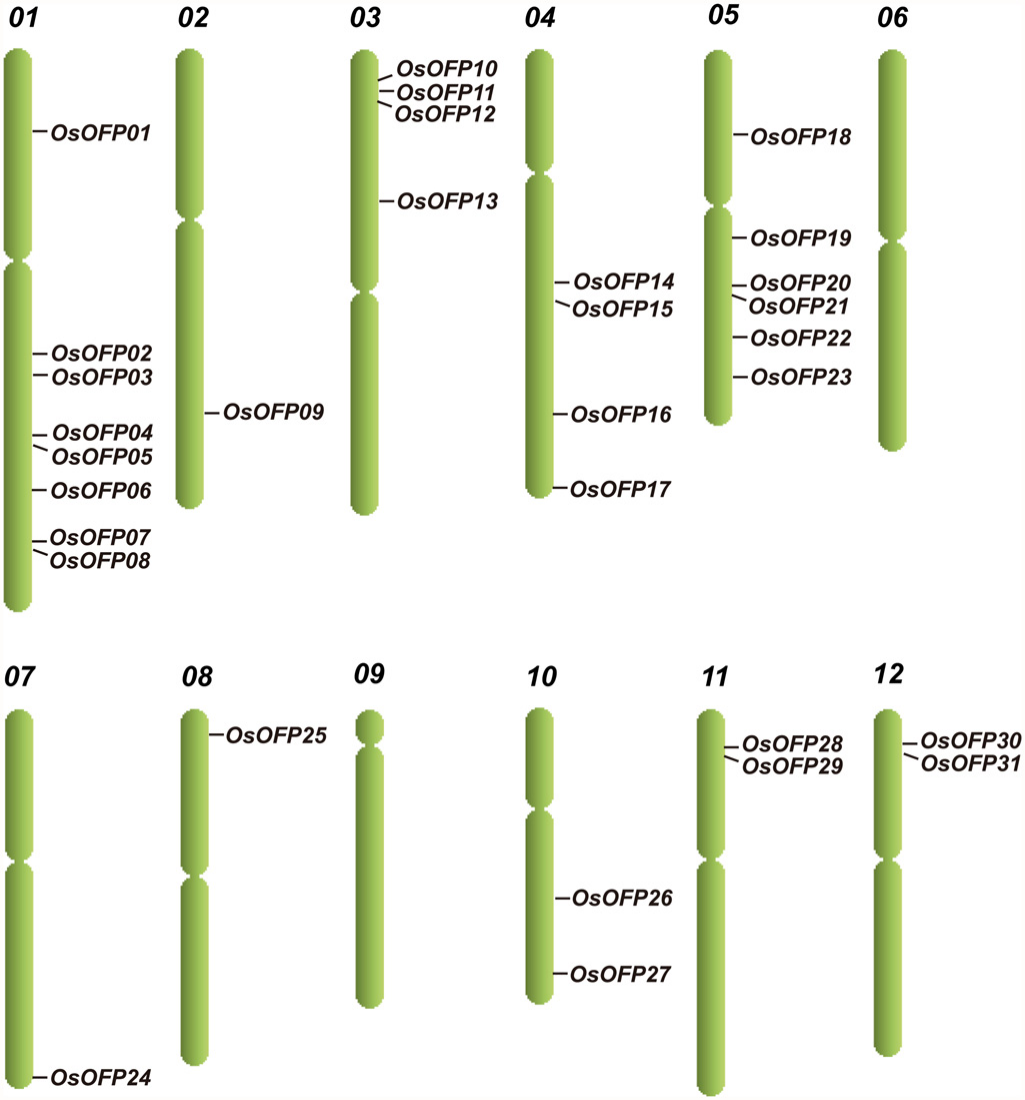
Genome distribution of the *OsOFP*s.

Thirty-one putative *OsOFP*s were identified in rice genome by searching the plant transcription factors database [20,21]. All 31 putative *OsOFP*s were designated as *OsOFP01*–*OsOFP*31 in accordance with their locations on the chromosomes of rice (Table 1a). Fig. 1 is a diagrammatic representation of the distribution of *OsOFP*s on the rice chromosomes. Although the *OsOFP*s are scattered throughout the 12 chromosomes of rice (except on chromosomes 6 and 9), their distribution is not uniform (Table 1b). The chromosomal location of the *OsOFP*s reveals that certain chromosomes and chromosomal regions have a relatively high density of *OsOFP*s. For instance, there are 8 and 6 *OsOFP*s located on chromosomes 01 and 05, respectively; however, only a single *OsOFP* was present on chromosomes 07 and 08, separately (Fig. 1).

**Table 1:** Information related to the OsOFPs, and performance of the ovate family protein.

Locus name	Gene name^a^	Chromosome location^b^	WoLF PSORT	CELLO	Subcellular localization
			Prediction^c^	Prediction^d^	Experiments^e^
**LOC_Os01g12690.1**	***OsOFP01***	**Chr1: 7009033–7010505**	**nucl(13)**	**Nuclear(4.3)**	**Nuclear**
**LOC_Os01g40970.1**	***OsOFP02***	**Chr1: 23173640–23174380**	**nucl(11.5) cyto_nucl(6.5) chlo(1)**	**Nuclear(3.3)**	**Nuclear with granular spots**
**LOC_Os01g43610.1**	***OsOFP03***	**Chr1: 24982666–24981173**	**nucl(14)**	**Nuclear(4.6)**	**Nuclear with granular spots**
**LOC_Os01g53160.1**	***OsOFP04***	**Chr1: 30542800–30544190**	**chlo(11) nucl(3)**	**Nuclear(3.6)**	**Nuclear with granular spots**
**LOC_Os01g54570.1**	***OsOFP05***	**Chr1: 31391261–31392235**	**nucl(13)**	**Nuclear(3.7)**	**Nuclear**
**LOC_Os01g60810.1**	***OsOFP06***	**Chr1: 35169295–35167600**	**chlo(7) nucl(2) mito(2) cyto(1) plas(1)**	**Nuclear(3.4)**	**Nuclear with granular spots**
**LOC_Os01g64410.1**	***OsOFP07***	**Chr1: 37381457–37382214**	**chlo(6) mito(6) plas(1)**	**Chloroplast(3.1)**	**Nuclear**
**LOC_Os01g64430.1**	***OsOFP08***	**Chr1: 37397573–37396145**	**nucl(7) chlo(6)**	**Nuclear(3.6)**	**Nuclear**
**LOC_Os02g45620.1**	***OsOFP09***	**Chr2: 27751407–27752436**	**chlo(8) nucl(3) mito(2)**	**Nuclear(3.1)**	**Nuclear**
**LOC_Os03g03480.1**	***OsOFP10***	**Chr3: 1506050–1505340**	**chlo(7) mito(6)**	**Nuclear(4.3)**	**Nuclear with granular spots**
**LOC_Os03g06350.1**	***OsOFP11***	**Chr3: 3179590–3178092**	**chlo(8) mito(4) nucl(2)**	**Nuclear(2.5)**	**Nuclear**
**LOC_Os03g10150.1**	***OsOFP12***	**Chr3: 5152038–5150966**	**chlo(12) mito(2)**	**Nuclear(2.9)**	**Nuclear**
**LOC_Os03g21870.1**	***OsOFP13***	**Chr3: 12510664–12509516**	**chlo(6) mito(6) nucl(1)**	**Nuclear(4.4)**	**Nuclear**
**LOC_Os04g33870.1**	***OsOFP14***	**Chr4: 20519152–20520509**	**chlo(8) nucl(2) mito(2) plas(1)**	**Chloroplast(3.4)**	**Nuclear**
**LOC_Os04g37510.1**	***OsOFP15***	**Chr4: 22333107–22332676**	**chlo(11) mito(2)**	**Nuclear(1.9) Extracellular(1.3)**	**Nuclear with granular spots**
**LOC_Os04g48830.1**	***OsOFP16***	**Chr4: 29121699–29123167**	**chlo(6) nucl(6) mito(1)**	**Nuclear(3.5)**	**Nuclear**
**LOC_Os04g58820.1**	***OsOFP17***	**Chr4: 34988834–34990126**	**nucl(14)**	**Nuclear(4.3)**	**Nuclear with granular spots**
**LOC_Os05g12808.1**	***OsOFP18***	**Chr5: 7357669–7356782**	**nucl(7) mito(5) chlo(2)**	**Chloroplast(1.9) Nuclear(1.4)**	**Nuclear**
**LOC_Os05g25910.1**	***OsOFP19***	**Chr5: 15070481–15069428**	**nucl(7) chlo(4) mito(3)**	**Nuclear(3.7)**	**Nuclear**
**LOC_Os05g36970.1**	***OsOFP20***	**Chr5: 21595856–21597025**	**chlo(7) nucl(6)**	**Nuclear(2.4)**	**Nuclear**
**LOC_Os05g36990.1**	***OsOFP21***	**Chr5: 21611604–21610390**	**nucl(5) mito(4) chlo(3) extr(1)**	**Nuclear(1.9)Chloroplast(1.3)**	**Nuclear**
**LOC_Os05g39950.1**	***OsOFP22***	**Chr5: 23477065–23478850**	**nucl(12) chlo(1)**	**Nuclear(4.3)**	**Nuclear**
**LOC_Os05g44090.1**	***OsOFP23***	**Chr5: 25636912–25635962**	**nucl(7) chlo(6)**	**Nuclear(4.0)**	**Nuclear**
**LOC_Os07g48150.1**	***OsOFP24***	**Chr7: 28759461–28760724**	**chlo(9) mito(3.5) cyto_mito(2.5)**	**Nuclear(3.4)**	**Nuclear**
**LOC_Os08g01190.1**	***OsOFP25***	**Chr8: 138247–139410**	**chlo(13)**	**Nuclear(3.4)**	**Nuclear**
**LOC_Os10g29610.1**	***OsOFP26***	**Chr10:15392044–15392805**	**chlo(11) mito(2)**	**Chloroplast(2.7)**	**Nuclear**
**LOC_Os10g38880.1**	***OsOFP27***	**Chr10:20714505–20715633**	**nucl(13)**	**Nuclear(2.8)**	**Nuclear**
**LOC_Os11g05770.1**	***OsOFP28***	**Chr11: 2659043–2660499**	**chlo(9) mito(2) nucl(1) cyto(1)**	**Chloroplast(2.0)**	**Nuclear**
**LOC_Os11g05780.1**	***OsOFP29***	**Chr11: 2666529–2665214**	**nucl(7) chlo(6)**	**Nuclear(4.0)**	**Nuclear**
**LOC_Os12g06150.1**	***OsOFP30***	**Chr12: 2898691–2900071**	**nucl(5) mito(5) chlo(3)**	**Nuclear(2.4)**	**Nuclear**
**LOC_Os12g06160.1**	***OsOFP31***	**Chr12: 2912589–2911273**	**nucl(9) chlo(3) mito(2)**	**Nuclear(3.3)**	**Nuclear**

(a) Rice *OFPs* were designated as *OsOFP01*–*OsOFP31*.

(b) Position of gene in the genomic fragment.

(c) The prediction from the subcellular localization program WoLF PSORT. The numbers in parenthesis indicate prior possible protein localization sites of the OsOFPs. Abbreviations of protein localization sites in the dataset are as follows: nucl: nucleus; chlo: chloroplast; cyto: cytosol; cysk: cytoskeleton; chlo_mito: chloroplast and mitochondria; plas: plastids; cyto_nucl: cytosol and nucleus; mito: mitochondria; pero: peroxisomes; and nucl_ plas: nucleus and plastids.

(d) The subcellular localization prediction program CELLO was used to determine the possible localization sites of the OsOFPs.

(e) Subcellular localization of the OsOFPs determined *via* the experiment in this study.

**Fig 2:**
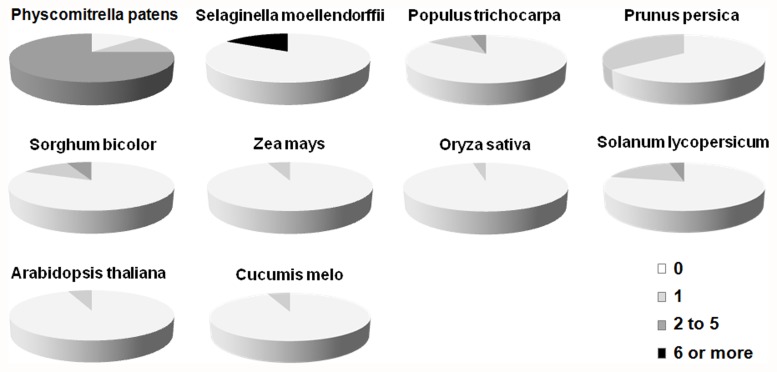
Relative proportions of intron-containing *OFP*s in *Physcomitrella patens*, *Selaginella moellendorffii*, *Populus trichocarpa*, *Prunus persica*, *Sorghum bicolor*, *Zea mays*, *Oryza sativa*, *Solanum lycopersicum*, *Arabidopsis thaliana* and *Cucumis melo*. Proportions are colored as: no introns (white), 1 intron (light gray), 2–5 introns (dark gray), and ≥6 (black).

*OFP*s are found in many species, from the moss to angiosperm, including *Physcomitrella patens*, *Selaginella moellendorffii*, *Populus trichocarpa*, *Prunus persica*, *Sorghum bicolor*, *Zea mays*, *Oryza sativa*, *Solanum lycopersicum*, *Arabidopsis thaliana* and *Cucumis melo*. The sequences of *OFP* genes from *Physcomitrella patens*, *Selaginella moellendorffii*, *Sorghum bicolor*, *Zea mays*, *Oryza sativa* and *Arabidopsis thaliana* were downloaded from PlnTFDB (http://plntfdb.bio.uni-potsdam.de/v3.0/). The *OFP* sequences of *Populus trichocarpa* and *Prunus persica* were blasted from Phytozome v10.0.2 (http://phytozome.jgi.doe.gov/pz/portal.html). *Solanum lycopersicum OFP* sequences were obtained from the tomato WGS Chromosomes (SL2.40) (SGN http://solgenomics.net) and from the reference published by Huang et al. (2013). The sequences of the *Cucumis melo OFP* were identified and downloaded through the MELONOMICS website (http://melonomics.net). Gene model and locus information (except the same and incomplete gene sequences) for these *OFP* family genes were listed in S1a-f Table and S2a-d Table. Using the online tool Gene Structure Display Server [22], we found that *OFP*s in angiosperm plants are inclined to be intron poor. Fig. 2 displays the proportions of *OFP*s in each species with no introns, 1 intron, 2–5 introns, and ≥6 introns. The final results indicate that 89, 25, 15, 36, 17, 6, 3, 26, 6 and 6% of the total number of *OFP*s in *P. patens*, *S. moellendorffii*, *Populus*, *P. persica*, *S. bicolor*, *Z. mays*, *O. sativa*, *S. lycopersicum*, *A. thaliana* and *C. melo* contain introns, respectively. In the *OsOFP* family, all 31 members (except *OsOFP14*) do not contain introns. Seven members in *SlOFP* family are single intron-containing genes. *SlOFP06* contains 2 introns [15]. No more than 2 introns appear in the intron-containing *OFP*s in *Populus*, *P. persica*, *Z. mays*, *O. sativa*, *S. lycopersicum*, *A. thaliana* and *C. melo*, even though the proportions of the *OFP*s with introns in *P. persica* and *S. lycopersicum* are higher than those in *S. moellendorffii*. In *P. patens* and *S. moellendorffii*, more introns were found in the intron-containing *OFP*s.

### Multiple Sequence Alignment and Phylogenetic Analysis of the OFP Proteins

**Fig 3:**
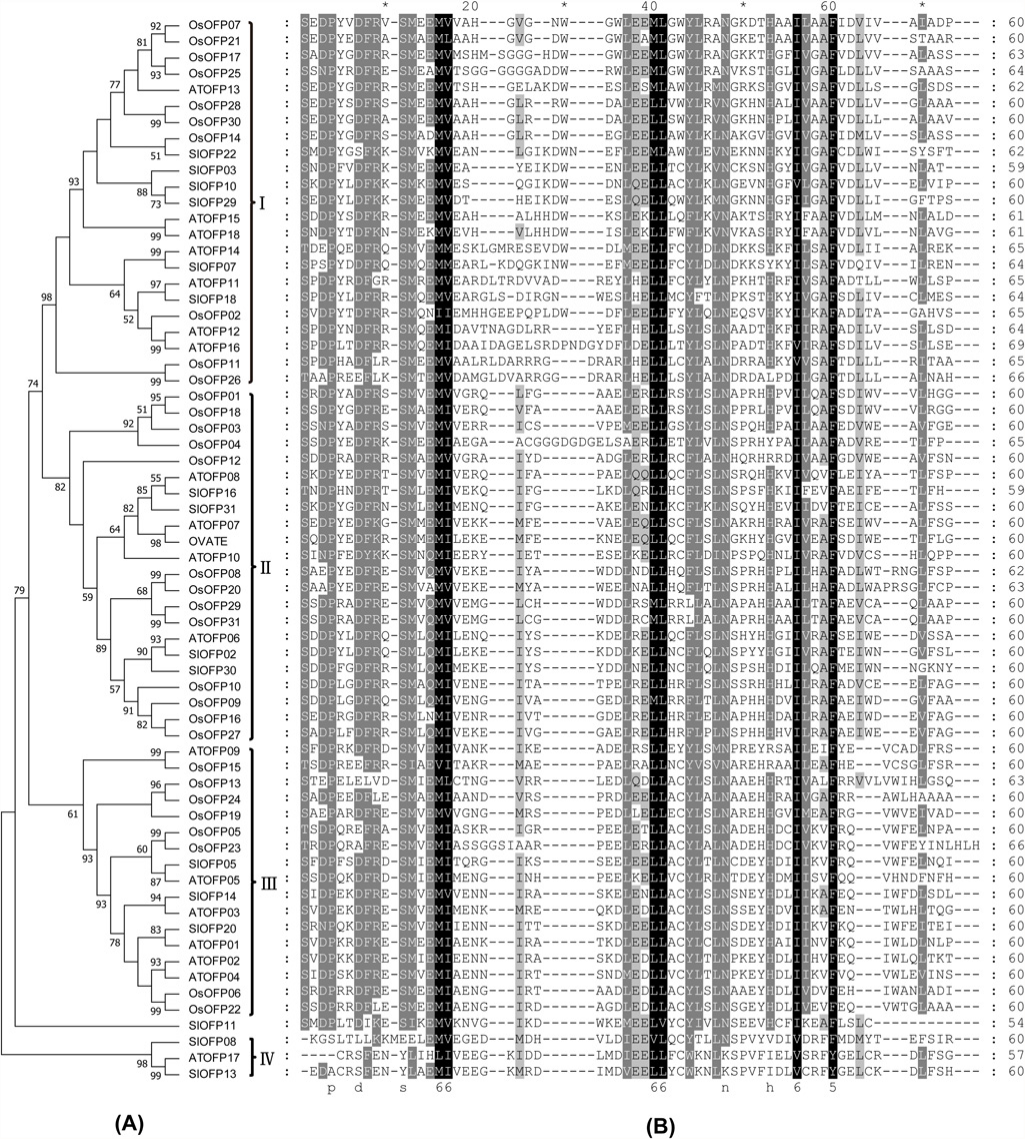
Multiple sequence alignment and phylogenetic tree of the OVATE domains of OFP proteins from rice, Arabidopsis and tomato. (A) The phylogenetic trees based on the multiple sequence alignments of the OVATE domains of OFP proteins. Bootstrap values from 1000 replicates are indicated at each node. The proteins on the tree can be divided into four distinct subfamilies (I–IV). Bootstrap values under 50% was not reported. (B) Multiple sequence alignments of the OVATE domains of rice, Arabidopsis and tomato are shown. The black region represents identical amino acids, and the grey region represents similar amino acids.

To investigate the phylogenetic relationship among rice, Arabidopsis and tomato *OFP* genes, the phylogenetic tree was constructed based on their OVATE domain sequences (Fig. 3A). The aligned OVATE domain sequences included 31 OVATE domains from rice, 18 OVATE domains from Arabidopsis and 17 OVATE domains from tomato (Fig. 3B). As shown in Fig. 3, excluding SlOFP11, all the other OFPs was further divided into 4 major subfamilies, designated I to IV. Except the smallest subfamily IV, each subfamily contains OFP members from rice, Arabidopsis and tomato. However, most of the OsOFP members were clustered in species-specific distinct clades. Only two pairs of orthologs, OsOFP15 and AtOFP09 between rice and Arabidopsis, OsOFP14 and SlOFP22 between rice and tomato could be figured out, comparing with 9 pairs of orthologs presented between Arabidopsis and tomato. These results suggest that the main characteristics of plant OFP proteins in rice, Arabidopsis and tomato were formed before divergence between monocots and dicots, and then evolved separately in a species-specific manner.

### Expression Patterns of *OsOFP*s

**Fig 4:**
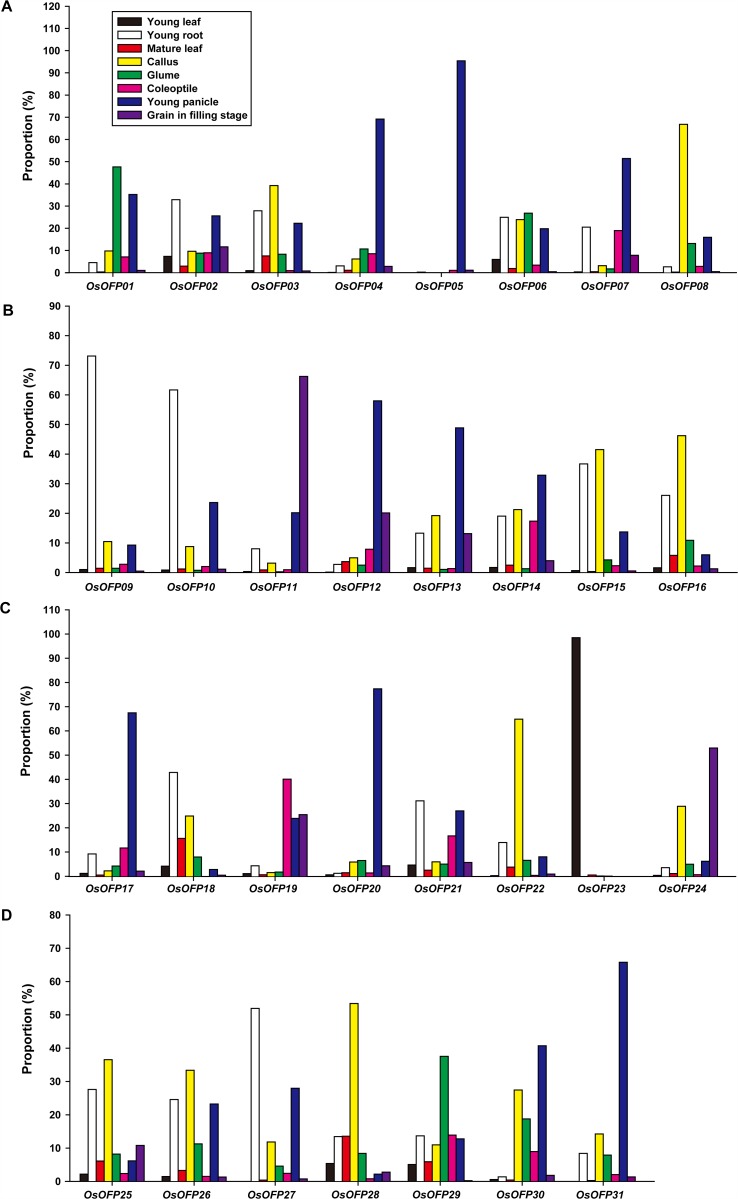
Expression profiles of the *OsOFP*s in various rice tissues at different developmental stages. The tissue-specific expression profiles were performed by qRT-PCR. The expression profiles of genes from *OsOFP01* to *OsOFP08* (A), The expression profiles of genes from *OsOFP09* to *OsOFP16* (B), The expression profiles of genes from *OsOFP17* to *OsOFP24* (C), The expression profiles of genes from *OsOFP25* to *OsOFP31* (D).

Gene expression pattern provides important clues for investigating gene function. The tissue-specific expression profiles of the 31 *OsOFP*s were performed by quantitative real-time RT-PCR (qRT-PCR). Total RNA was isolated from calli, coleoptiles, young leaves, young roots, mature leaves, glumes, young panicles and grains in filling stage (Fig. 4). The results showed that *OsOFP*s exhibited different expression patterns. The transcript levels of *OsOFP01*, *OsOFP06* and *OsOFP29* were higher in the glumes than in other organs (Fig. 4). *OsOFP02*, *OsOFP09*, *OsOFP10*, *OsOFP18*, *OsOFP21* and *OsOFP27* were prominently higher in the roots than in other organs (Fig. 4). *OsOFP03*, *OsOFP08*, *OsOFP15*, *OsOFP16*, *OsOFP22*, *OsOFP25*, *OsOFP26* and *OsOFP28* were obviously higher in the calli than in other organs (Fig. 4). The expression patterns of *OsOFP04*, *OsOFP05*, *OsOFP07*, *OsOFP12*, *OsOFP13*, *OsOFP14*, *OsOFP17*, *OsOFP20*, *OsOFP30* and *OsOFP31* were all very similar, and they were preferentially expressed in the young panicles tissue (Fig. 4). *OsOFP11* and *OsOFP24* were more highly expressed in the grains of filling stage but were only minimally expressed in other organs (Fig. 4B, 4C). The levels of *OsOFP19* were notably expressed in the coleoptiles (Fig. 4C). *OsOFP23* was abundantly expressed in the young leaves but was minimally expressed in other tissues (Fig. 4C). Interestingly, ~50% of the total number of *OsOFP*s was more highly expressed during the seed developmental stage than that during other stages of development. This is similar to the expression profile of the tomato *OVATE* which is expressed in reproductive organs in early stages of flower and fruit development.

The seed developmental cis-elements that have been well characterized were investigated in 2-kb 5′-upstream regions of the *OsOFP*s [23]. The GCN4 motif is found in the promoter region of the glutelin gene, which encodes a major seed storage protein and is involved in endosperm-specific gene expression in rice [24–26]. The Skn-1 motif is a consensus sequence in a number of seed-specific promoters and is associated with endosperm-specific gene expression [24]. The promoter region of each *OsOFP* contains at least one of the 2 seed development *cis*-elements (S1 Fig.).

### Hormone-Induced Expression Profiles of *OsOFP*s

*AtGA20OX1* is thought to be a direct target gene of *AtOFP1* and exogenous gibberellic acid can partially rescue the reduced length in rapidly elongating aerial organs of plants overexpressing *AtOFP1*, suggesting its important role in suppressing GA biosynthesis and maintaining the balance of this hormone in plants [11]. The expression profiles of *OsOFP*s responding to exogenous GA3 were investigated in our study. Transcript levels of *OsOFP*s did not change significantly in response to GA treatment (S2 Fig.), indicating that *OsOFP*s may not participate in the response to GA in rice.

**Fig 5:**
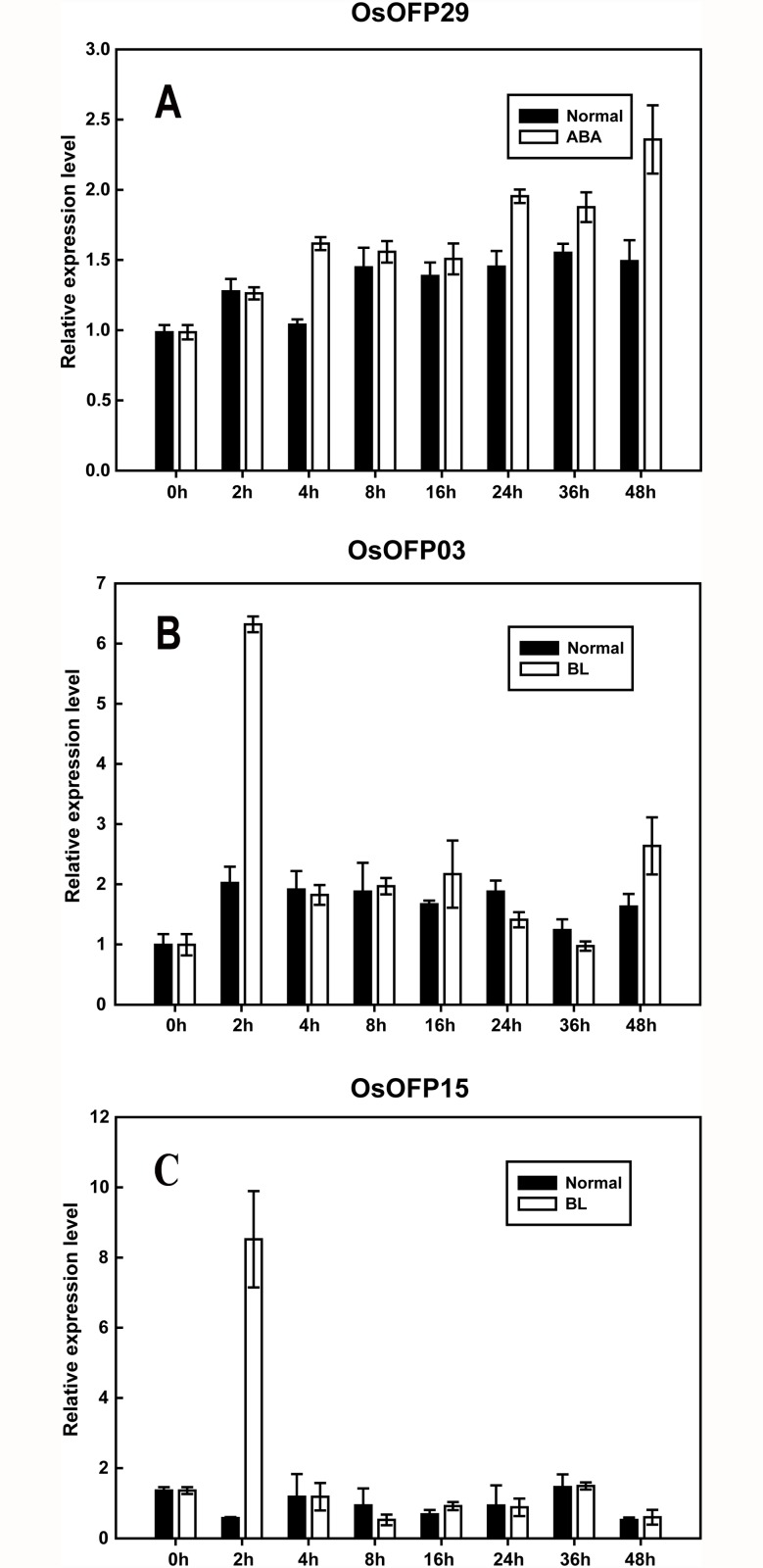
Expression profiles of *OsOFP*s under phytohormone treatments. (A) Expression profile of *OsOFP*29 responding to ABA (50 μM; 0, 2, 4, 8, 16, 24, 36, and 48 h). (B, C) Expression profiles of *OsOFP03* and *OsOFP15* responding to BR, respectively (1 μM; 0, 2, 4, 8, 16, 24, 36, and 48 h). The expression profiles of *OsOFP*s responding to ABA and BL were determined by qRT-PCR. Actin was used as an internal control. Error bars indicate the SD based on 3 biological replicates.

In order to further characterize the relevance between *OsOFP* family and hormone signaling, the expression patterns of the *OsOFP* family in response to abscisic acid (ABA) and brassinosteroid (BR), the 2 classes of hormones whose essential function in plant development and stresses resistance were widely appreciated [27–33], were also assessed using the quantitative real-time RT-PCR (qRT-PCR) in the current study (Fig. 5; S3 Fig., S4 Fig.). The results showed that only *OsOFP29* was upregulated after 24 h of the ABA treatment (Fig. 5A), whereas the transcript levels of other *OsOFP*s did not change significantly (S3 Fig.). Interestingly, *OsOFP03* and *OsOFP15* were upregulated immediately under the brassinolide (BL) treatment. The transcript level of *OsOFP03* increased quickly and reached its maximum at 2 h (Fig. 5B). *OsOFP15* exhibited a response similar to that of *OsOFP03* under the BL treatment (Fig. 5C). The quick responses of the *OsOFP*s to the BL treatment indicate that they may act as important upstream components in BL signal transduction in rice.

### Localization of OsOFP Proteins

A previous study has shown that ovate proteins are transcriptional repressors in *Arabidopsis* [11], but little is known about the molecular function and subcellular localization of OVATE proteins in rice. Usually, transcription regulators are localized in the nucleus to turn a group of target genes on or off. Most of the known AtOFPs are active in the nucleus [9–12]. The predicted target signal peptides for OVATE proteins in rice were checked using the WoLF PSORT [34] and CELLO [35] prediction programs. According to both programs, most proteins in this family were assigned to either the nucleus or chloroplast (Table 1c, d).

Using the WoLF PSORT program, the OsOFPs were predicted to locate in the nucleus or chloroplast (Table 1c). CELLO indicated that most OsOFPs scored relatively higher in the nucleus than in the chloroplast (Table 1d). In total, the differences in the location of OsOFP10, OsOFP12, OsOFP15, OsOFP24, and OsOFP25 were notable between the two prediction programs (Table 1c, d). Although not conclusive, the presented bioinformatics data could serve as a useful reference for further studies on OVATE protein functions in plants.

**Fig 6:**
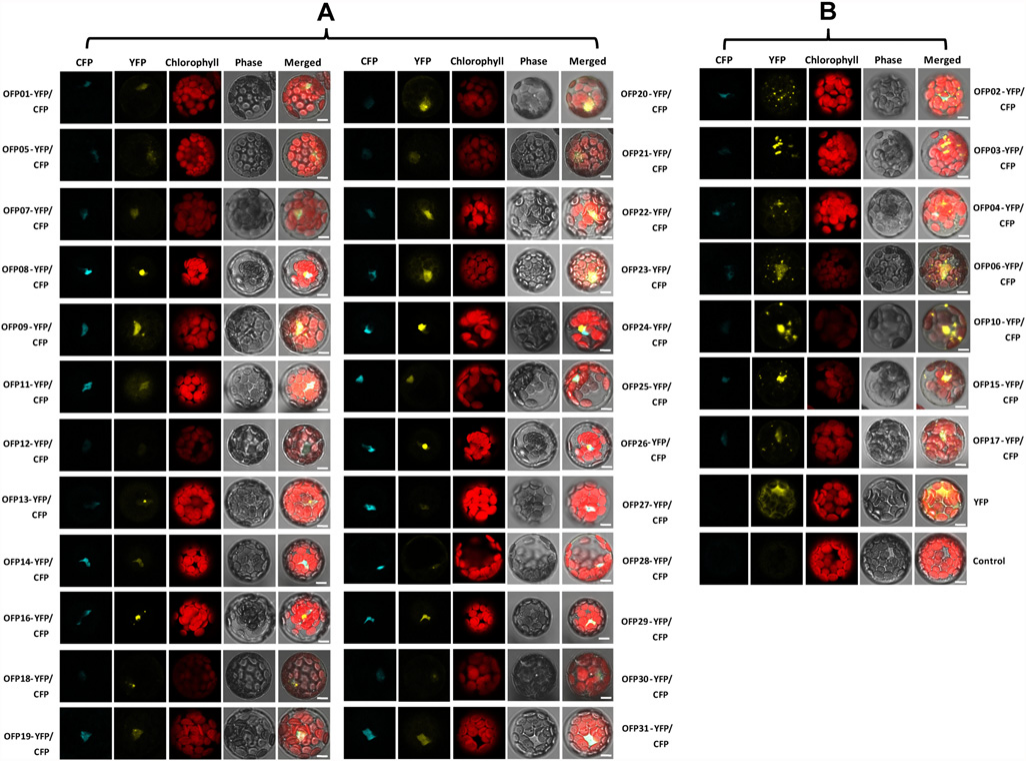
Subcellular localization of the OsOFPs. Protoplast transient expression using YFP-OsOFP fusion constructs were used to determine the subcellular localization. AHL22 fused with CFP was used as a nuclear marker. The 35S: YFP-OsOFP and 35S: CFP-AHL22 constructs were transformed into an *Arabidopsis* protoplast cell. Fluorescence images of CFP, YFP, and chlorophyll autofluorescence (Chl) were captured with confocal laser scanning microscopy and are shown in cyan, yellow, and red, respectively (scale bars, 10 μm).

To confirm the subcellular localization of the OsOFP family, the co-localization of OsOFP yellow fluorescent proteins (YFP) and the nuclear marker AHL22-cyan fluorescent protein [36] were analyzed. The corresponding full-length coding sequences were systematically cloned into a pDONR/Zeo gateway entry vector. This recombined cloning system was subsequently used to further subclone *OsOFP* coding sequences into the YFP fused destination vectors. As shown in Fig. 6A, 24 OsOFPs were shown to target in the nucleus, and the others were shown to target in the nucleus with granular spots (Fig. 6B). The results were, in general, consistent with the bioinformatics prediction of the subcellular localization of OsOFPs (Table 1c, d, e).

## Discussion

OVATE family proteins were present in 13 sequenced plant genomes that represent the major evolutionary lineages of land plants [37]. During the course of plant evolution, most gene families expanded mainly through large scale segmental duplication or tandem duplication to maintain the large number of family members [38], manner of gene duplication had been described in many large transcription factor families such as the *bHLH* family [39] and the *bZIP* family [40] in rice. In this study, the relatively high density chromosomal distribution pattern of the *OsOFP*s indicates that large-scale segmental duplication or tandem duplication may have been involved in the expansion of the *OsOFP* family. A recent study had verified that segmental duplication and tandem duplication were two major mechanisms for gene expansion in the OsOFP family. For instance, 24 (72.7%) were located within segmental duplication blocks and eight (24.2%) were tandemly duplicated in the rice OsOFP family [37].

Previous studies have shown that OFPs function as transcriptional repressors and regulate multiple aspects of plant growth and development [9,11–13,41]. The *OVATE* gene determines the conversion of the tomato fruit from round- to pear-shaped, and overexpression of this gene results in smaller-sized floral organs and leaflets [13]. The tomato *OVATE* transcripts can be detected in flowers 10 days before anthesis and until 8 days after anthesis in developing fruit [13]. Recently, Huang et al. (2013) reported that the *OVATE* expression increased in fruit at 33 days post of anthesis, suggesting its important role at the fruit ripening stage [15]. To some extent, there is coincidence between the spatially restricted expression of *OVATE* and the pear-shaped fruit shape phenotype [13]. Round seeds or blund-end siliques were also observed in *AtOFP* family members overexpressed *A. thaliana* respectively [11,12]. Furthermore, an Ovate-like gene in pepper was reported to be involved in determining fruit shape [41]. In this study, tissue-specific expression pattern analysis showed that *OsOFP*s were expressed in all developmental phases of the rice plant, and nearly half of this gene family was more highly expressed during the seed developmental stage (Fig. 4). Moreover, each *OsOFP* contained the GCN4 or Skn-1 motifs, 2 of the seed developmental cis-elements that have been well characterized in rice, in the promoter region (S1 Fig.). Results from the functional analysis of the *OFP*s in the above plant species and from the current study hint at key roles of *OsOFP*s in controlling important traits related to seed development in rice.

TFs function as key regulators during different stages of seed development [42–44]. A pair of basic helix-loop-helix (bHLH) proteins, PGL1 and APG, are involved in determining grain weight and length antagonistically, in which APG is a negative regulator whose function is inhibited by PGL1 [42]. The homeobox family of genes was found to play a pivotal role throughout the process of seed development. For instance, homeobox gene *OSH1* is critical for the regionalization of cell identity during early embryogenesis [43]. Studies in *Arabidopsis* showed that AtOFPs interact with TALE homeodomain proteins to regulate plant development [9]. Results of most *OsOFP*s with stronger expression profiles in the mature seed or embryo suggest that *OsOFP*s may act as important transcriptional regulators during seed development in rice. Further studies are needed to identify the interactions of OsOFPs in order to discover new seed development regulating pathways that include OsOFP at their core.

Many transcriptional factor families were characterized to play important roles in different developmental processes regulated by phytohormones [45–47]. So far, only AtOFP1 was reported to target *AtGA20ox1*, a key enzyme gene in gibberellin biosynthesis, and to suppress its expression in plants overexpressing *AtOFP1* [9,11]. No information about the relevance between plant OFP families and the other phytohormones was provided. In this study, the expression profiles of *OsOFP*s responding to exogenous GA3, ABA and BL were investigated (Fig. 5; S2 Fig., S3 Fig., S4 Fig.). We found that the transcript level of *OsOFP15* increased rapidly in response to BL treatment, meanwhile it was specifically expressed in the mature seed. Brassinolide is the most bioactive form of the growth-promoting plant steroid hormones, termed brassinosteroids. Brassinosteroids are essential for a wide range of developmental and physiological processes [28–31]. BR-deficient and BR-insensitive mutants display dwarfism, erect leaves, and altered panicle length in combination with a reduced seed setting [48–50]. XIAO, a putative LRR receptor-like kinase, is related to BR biosynthesis; and the loss-of-function mutant, *xiao*, leads to reduced plant size and seed setting due to a decrease in the rate of cell division [49]. Overexpression of the BR biosynthesis related gene *Zm-CYP-1* increased grain yield by controlling the seed filling in rice [31]. The spatially restricted and BR-induced expression pattern of *OsOFP15* suggest that it may participate in regulating the BR response in a rice seed setting.

Subcellular proteomics are helpful in exploring the biological functions of proteins. In the current study, the subcellular localization analysis clearly revealed that the YFP-OsOFP fusion proteins predominantly localized in the nucleus. With regard to OsOFP05, 28, and 30, only very weak yellow fluorescent signals were observed in the nucleus (Fig. 6), while strong yellow fluorescent signals were clearly observed in the nucleus for other OsOFPs (Fig. 6). The reason for these observations may be that OsOFP05, 28, and 30 were not stable when compared to the other OsOFPs in the nucleus of *Arabidopsis* protoplast cells. We suspected that the N-terminus-fused YFP may be the reason for the instability of the OsOFPs with weak fluorescent signals. As exhibited in Fig. 6, OsOFP02, 03, 04, 05, 06, 10, 15, and 17 prominently displayed nuclear concentrations with multiple big and small granular spots floating along the nucleus. Some transcription factor proteins were nuclear localization and also present in the cytoplasm [51]. For instance, the KNOX proteins, encoding transcription factors involved in shoot apical meristem development and maintenance, had similar subcellular distribution patterns to these OsOFP proteins [52]. BZR1 and BZR2/BES1, the two key transcription factors of the BR signaling pathway, were found in both the nucleus and cytoplasm [53,54]. BR signal increased the nuclear accumulation of BZR1 and BZR2/BES1 [53–55]. The subcellular distrbution patterns of these OsOFPs suggested that they may function as potential nucleocytoplasmic shuttling proteins and can be regulated by various signals such as hormone and light [56].

The localization prediction programs for OsOFPs on the Website do not support the actual subcellular localization for some proteins. For example, OsOFP07, OsOFP10, OsOFP12, OsOFP14, OsOFP15, OsOFP24, OsOFP25, OsOFP26 and OsOFP28 did not localize in the nucleus according to the predictions of the WoLF PSORT or CELLO programs (Table 1c, d, e). However, OsOFPs were shown to target to the nucleus in the current study. This discrepancy may be explained by the fact that the nuclear localization signals identified and deposited in the WoLF PSORT and CELLO databases are not complete. It is also possible that novel nuclear localization signals, that have not yet been identified, could exist in the OsOFP sequences.

In conclusion, this study provided the genomic framework, intron number, and phylogenetic analysis of the 31 *OsOFP*s. In addition, we studied the expression profile of the whole family during the entire life cycle of the rice plant and under GA, ABA, and BL treatments. Furthermore, the subcellular localization of OsOFPs was also investigated. Our data provide insight into the roles of *OsOFP*s during the seed developmental stages and in response to BL. This study provides a useful reference for conducting more detailed functional analyses of these *OFP*s in rice and will be helpful in the selection of appropriate candidate genes for further studies.

## Materials and Methods

### Phylogenetic analysis and sequence alignment

Multiple sequence alignment were performed using the ClustalX version 1.83 [57] and were manually corrected. The obtained sequence alignments were used as input to construct phylogenetic tree with the neighbor—joining algorithm in MEGA 6.06 [58]. Bootstrap analysis was performed using 1000 replicates.

### Plant Material and Growth Conditions

Wild-type Nipponbare (*O. sativa* L. ssp. *japonica*) was used in the current study. The seeds were imbibed for 48 h at 30°C and cultivated in the test field or growth chamber under diurnal conditions (day phase: 10 h/30°C; night phase: 14 h/24°C). For tissue-specific expression analysis, materials were collected at different developmental stages. Young leaves, young roots, calli and coleoptiles were collected from the growth chamber; and mature leaves, glumes, young panicles and grains in filling stage were collected from the test field of Jilin University. All plant materials were collected in liquid nitrogen and stored at -80°C for RNA extraction.

*Arabidopsis* seeds of the Columbia ecotype (Col-0) were used in our study. Seeds were surface-sterilized, vernalized, and sown and grown on 1/2 MS media until the leaves reached the four-leaf stage. Then, the seedlings were transplanted to a growth chamber containing peat moss mixed with vermiculite (1/1, v/v), under diurnal conditions (day phase: 8 h/22°C; night phase: 16 h/18°C). The relative humidity was maintained at 60–70%. Leaves were collected from 3- to 5-week-old seedlings for protoplast isolation and transfection assays.

### RNA Isolation and cDNA Synthesis

Total RNA was extracted using the TRIzol reagent (Invitrogen, Carlsbad, CA, USA) according to the manufacturer’s instructions. The integrity of the RNA was checked electrophoretically, and complementary DNA (cDNA) was synthesized using 4 μg of RNA with the Oligo d(T)_18_ primers and M-MLV reverse transcriptase (TaKaRa Bio, Tokyo, Japan), according to the manufacturer’s protocol.

### Real-time Quantitative PCR Analysis

Relative transcript level expression profiles of *OsOFP*s were evaluated by real-time quantitative PCR (RT-qPCR) on Mx3005P (Stratagene, La Jolla, CA, USA) using the SYBR Premix ExTaq polymerase (TaKaRa, Bio Inc.). Each reaction contained 12.5 μl of the 2× SYBR Premix ExTaq, 50–100 ng of the cDNA template, 0.5 μl of 10 mM of each primer, and 10.5 μl of double-distilled H_2_O for a final volume of 25 μl. The PCR reaction parameters were 95°C for 30 s (1 cycle), 95°C for 5 s, and 60°C for 20 s (40 cycles), which was followed by a melting curve analysis at 95°C for 60 s, 55°C for 30 s, and 95°C for 30 s. The relative fold differences were calculated based on the comparative 2^-ΔΔ Ct^ method. Approximately 100–200-bp PCR products, unique to each *OsOFP*, were amplified; the housekeeping gene *ACTIN* (X15863.1) was used to normalize the transcript level of each *OsOFP* in the samples. Expression profile analysis of *OsOFP* genes in each type tissue was displayed using relative percentage [15] in this study. The specific primer pairs used were listed in S3 Table.

### Expression Profiles of *OsOFP*s in Response to ABA and BL

The rice seeds of the wild-type Nipponbare were sterilized with 3% NaOCl for 30 min, washed extensively with distilled water, and placed in petri dishes with wetted filter papers at 30°C in the dark. After 3 d of incubation, germinated seeds were sown in a 96-well plate, with the bottom removed, and the plate was fixed in a 1/2 MS culture solution at 26°C under continuous light. After 14 d, the seedlings were subjected to ABA and BL treatments. For the ABA or BL treatments, seedlings were transferred to a culture solution with 50 μM of ABA, 1 μM of BL, or without ABA and BL (i.e., the control). Subsequently, young leaves were collected at 0, 2, 4, 8, 16, 24, 36, and 48 h after treatment. Then the expression profiles of *OsOFP*s responding to ABA and BL were evaluated by real-time quantitative PCR (RT-qPCR).

### Construction of the Subcellullar Localization Plasmid

The expression vectors of subcellullar localization were constructed as follows: the coding sequences of the *OsOFP*s were cloned into an entry vector (pDONR/Zeo; Invitrogen) by using the BP-clonase, according to the manufacturer’s instructions, and subsequently cloned into the destination vector (pENSG-YFP) of the N-terminus fused the YFP reporter gene by an LR reaction (Gateway recombination, Invitrogen). All of the fusion constructs were driven by the 35S promoter. The recombinant plasmids vector pENSG-CFP-AHL22, carrying a nuclear marker gene AHL22 [36], was co-transformed with each target gene. The specific primer pairs used were listed in S4 Table.

### Protoplast Isolation, Transformation, and Confocal Microscopy

Leaves of 3- to 5-week-old plants were used in the subcellular localization analysis. The leaves were cut into 0.5–1-mm strips with fresh razor blades (without wounding) and incubated in an enzyme solution that included 1% cellulase R10, 0.25% macerozyme R10, 0.4 M mannitol, 20 mM KCl, 10 mM CaCl_2_, and 20 mM MES for 3–4 h, with 40–50 rpm slow shaking. After incubation, the protoplast suspension was filtered through a metal sieve and centrifuged at 100 × *g* for 5 min. The pelleted protoplasts were suspended in 5 mL of a W5 solution (154 mM NaCl, 125 mM CaCl_2_, 5 mM KCl, and 2 mM MES/KOH; pH 5.7) and centrifuged for 5 min at 100 × *g*. The protoplasts were transferred to a tube containing 5 mL of the W5 solution. The protoplasts were pelleted again by centrifugation at 100 × *g* for 5 min and resuspended in 5 mL of the W5 solution. The protoplasts were incubated on ice for 30 min. The protoplasts were again resuspended in 5 mL of MMg buffer (400 mM mannitol, 15 mM MgCl_2_, and 4 mM MES/KOH; pH 5.7).

For cotransformation, 10 μg of each plasmid DNA was added to 100 μL of the protoplast suspension. An equal volume of 40% (w/v) PEG3350, freshly prepared with 0.1 M CaCl_2_ and 0.8 M mannitol solution, was added. Then, the mixture was incubated at room temperature for 30 min. After incubation, the mixture was diluted with 500 μL of the W5 solution. The solution was fully mixed, and the protoplasts were pelleted by centrifugation at 100 × *g* for 5 min. Then, the protoplasts were washed twice using the W5 solution, resuspended gently in 1 mL of the W5 solution, and incubated in 12-well plates at room temperature for 18–20 h in darkness.

All microscopic observations were performed using a confocal laser scanning microscope. The fluorescence of the YFP was visualized with excitation and emission wavelengths of 488 and 505–530 nm, respectively. For CFP, the excitation and emission wavelengths were 458 and 465–530 nm, respectively. Chloroplast autofluorescence was visualized in a detection channel with excitation and emission wavelengths of 488 and 650–710 nm, respectively. Image processing was performed with ImageJ (http://rsb.info.nih.gov/ij/).

## Supporting Information

S1 Fig Distribution of major seed developmental cis-elements (GCN4 and Skn-1) in the 2-kb promoter region of the *OsOFP*s. Two figures were used to represent different seed developmental cis-elements; the lines represent promoter sequences. The elements located in the forward and reverse strands are indicated as ‘+’ and ‘-’, respectively.
(TIF)

S2 Fig Expression profile of 31 *OsOFP*s responding to GA. (TIF)

S3 Fig Expression patterns of 30 *OsOFP* genes in response to ABA treatments. (TIF)

S4 Fig Expression profile of 29 *OsOFP*s responding to BR. (TIF)

S1 Table Basic information about *OFP* family genes. (a, b, c, d, e, f) Gene model of *OFP* family genes in physcomitrella patens, selaginella, sorghum, arabidopsis, maize and rice, respectively.
(DOC)

S2 Table Basic information about *OFP* family genes. (a, b, c, d) Gene locus of *OFP* family genes in populus trichocarpa, prunus persica, solanum lycopersicum and cucumis melo, respectively.
(DOC)

S3 Table Primers for real-time quantitative PCR of *OsOFP*s. (DOC)

S4 Table Primers for systemic subcellular localization assays of OsOFPs. (DOC)
