# Supplementary figures and images for: Expression Pattern and Subcellular Localization of the Ovate Protein Family in Rice
Source: PLoS One. 2015 Mar 11;10(3):e0118966. doi: 10.1371/journal.pone.0118966 (PMC4356581; doi:10.1371/journal.pone.0118966)

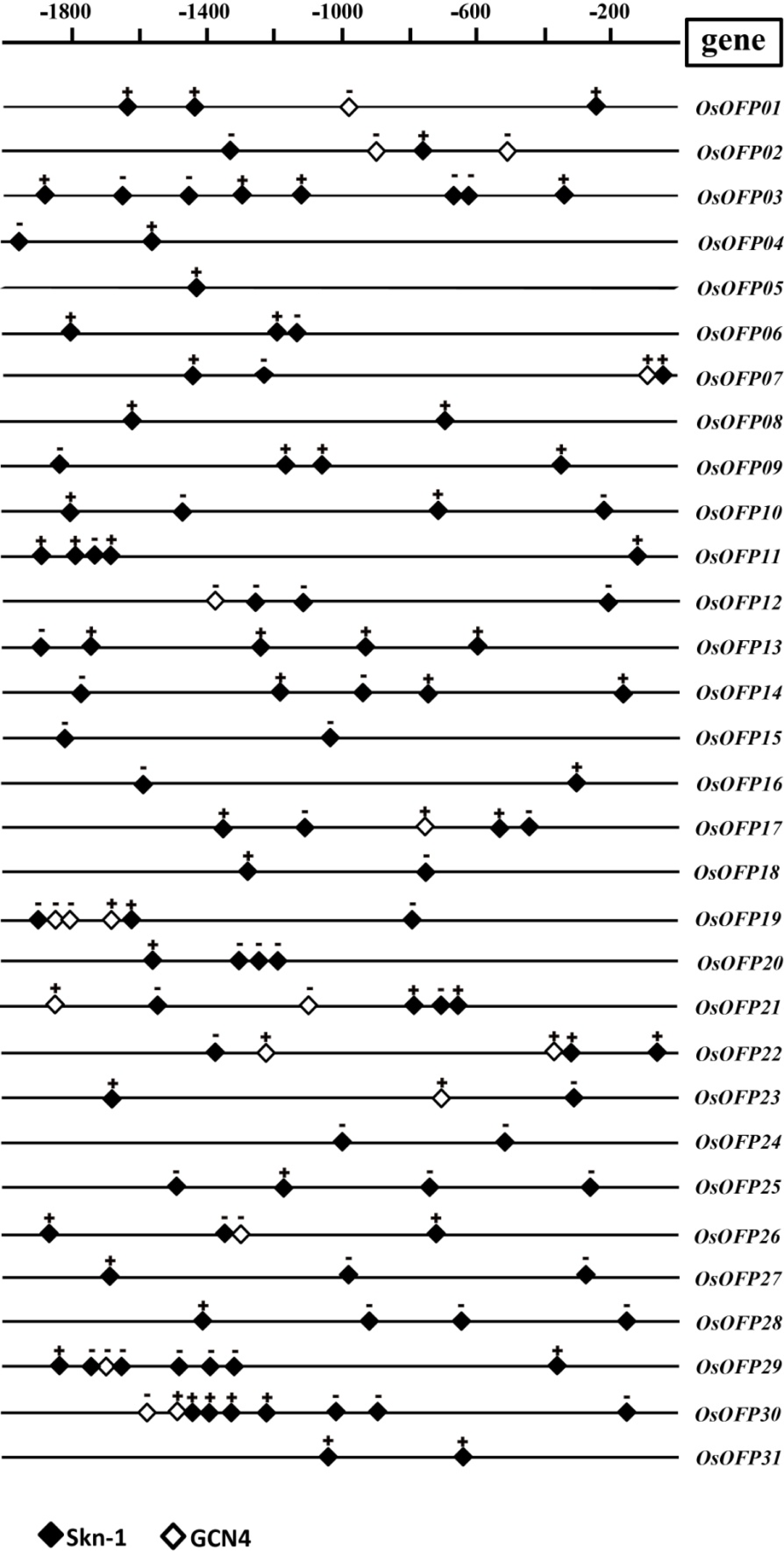

Supplement: S1 Fig — Two figures were used to represent different seed developmental cis-elements; the lines represent promoter sequences. The elements located in the forward and reverse strands are indicated as ‘+’ and ‘-’, respectively. (TIF) [file pone.0118966.s001.tif]
