# Supplementary material for: Expression Pattern and Subcellular Localization of the Ovate Protein Family in Rice
Source: PLoS One. 2015 Mar 11;10(3):e0118966. doi: 10.1371/journal.pone.0118966 (PMC4356581; doi:10.1371/journal.pone.0118966)
Supplement: S1 Table — (a, b, c, d, e, f) Gene model of OFP family genes in physcomitrella patens, selaginella, sorghum, arabidopsis, maize and rice, respectively. (DOC) [file pone.0118966.s005.doc]

**Table S1. Basic information about OFP family genes.**

| **Physcomitrella patens**  **(Gene model)^a^** | **Selaginella**  **(Gene model)^b^** | **Sorghum (Gene model)^c^** | **Arabidopsis (Gene model)^d^** | **Maize**  **(Gene model)^e^** | **Rice**  **(Gene model)^f^** |
| --- | --- | --- | --- | --- | --- |
| 229652 | 117843 | 125007 | AT1G05420.1 | AC191261.3_FGP041 | LOC_Os01g12690.1 |
| 64682 | 407613 | 126240 | AT1G06920.1 | AC196390.3_FGP022 | LOC_Os01g40970.1 |
| 65669 | 417707 | 148852 | AT1G79960.1 | GRMZM2G001721_T01_P01 | LOC_Os01g43610.1 |
| 70422 | 437676 | 4965680 | AT2G18500.1 | GRMZM2G013271_P01 | LOC_Os01g53160.1 |
| 77394 |  | 4970590 | AT2G30400.1 | GRMZM2G013302_P01 | LOC_Os01g54570.1 |
| 87111 |  | 4975699 | AT2G32100.1 | GRMZM2G023285_P01 | LOC_Os01g60810.1 |
| 95573 |  | 4976435 | AT2G36026.1 | GRMZM2G026927_P01 | LOC_Os01g64410.1 |
| 98835 |  | 4983989 | AT2G36050.1 | GRMZM2G027865_P01 | LOC_Os01g64430.1 |
|  |  | 4985062 | AT3G52525.1 | GRMZM2G028982_P01 | LOC_Os02g45620.1 |
|  |  | 4988638 | AT3G52540.1 | GRMZM2G032478_P01 | LOC_Os03g03480.1 |
|  |  | 4988639 | AT4G04030.1 | GRMZM2G037043_P01 | LOC_Os03g06350.1 |
|  |  | 4991063 | AT4G14860.1 | GRMZM2G039312_P01 | LOC_Os03g10150.1 |
|  |  | 4991233 | AT4G18830.1 | GRMZM2G040673_P01 | LOC_Os03g21870.1 |
|  |  | 4991841 | AT5G01840.1 | GRMZM2G041761_P01 | LOC_Os04g33870.1 |
|  |  | 4992019 | AT5G04820.1 | GRMZM2G044013_T01_P01 | LOC_Os04g37510.1 |
|  |  | 4992262 | AT5G19650.1 | GRMZM2G053656_P01 | LOC_Os04g48830.1 |
|  |  | 4998128 | AT5G22240.1 | GRMZM2G053985_P01 | LOC_Os04g58820.1 |
|  |  | 4998989 | AT5G58360.1 | GRMZM2G055257_P01 | LOC_Os05g12808.1 |
|  |  | 5002211 |  | GRMZM2G055737_P01 | LOC_Os05g25910.1 |
|  |  | 5029664 |  | GRMZM2G057753_P01 | LOC_Os05g36970.1 |
|  |  | 5029715 |  | GRMZM2G057794_P01 | LOC_Os05g36990.1 |
|  |  | 5030332 |  | GRMZM2G067376_P01 | LOC_Os05g39950.1 |
|  |  | 5030460 |  | GRMZM2G067931_P01 | LOC_Os05g44090.1 |
|  |  | 5034864 |  | GRMZM2G068036_P01 | LOC_Os07g48150.1 |
|  |  | 5036149 |  | GRMZM2G075988_P01 | LOC_Os08g01190.1 |
|  |  | 5036647 |  | GRMZM2G078626_P01 | LOC_Os10g29610.1 |
|  |  | 5036961 |  | GRMZM2G092035_P01 | LOC_Os10g38880.1 |
|  |  | 5038947 |  | GRMZM2G095452_P01 | LOC_Os11g05770.1 |
|  |  | 5039560 |  | GRMZM2G096252_P01 | LOC_Os11g05780.1 |
|  |  | 5041920 |  | GRMZM2G100133_P01 | LOC_Os12g06150.1 |
|  |  | 5042786 |  | GRMZM2G105444_P01 | LOC_Os12g06160.1 |
|  |  | 5042826 |  | GRMZM2G105876_P01 |  |
|  |  |  |  | GRMZM2G106110_P01 |  |
|  |  |  |  | GRMZM2G106781_P01 |  |
|  |  |  |  | GRMZM2G106836_P01 |  |
|  |  |  |  | GRMZM2G120283_P01 |  |
|  |  |  |  | GRMZM2G121706_P01 |  |
|  |  |  |  | GRMZM2G122585_P01 |  |
|  |  |  |  | GRMZM2G122709_P01 |  |
|  |  |  |  | GRMZM2G124334_P01 |  |
|  |  |  |  | GRMZM2G127431_P01 |  |
|  |  |  |  | GRMZM2G127680_P01 |  |
|  |  |  |  | GRMZM2G129532_P01 |  |
|  |  |  |  | GRMZM2G130131_P01 |  |
|  |  |  |  | GRMZM2G132922_P01 |  |
|  |  |  |  | GRMZM2G133311_P01 |  |
|  |  |  |  | GRMZM2G137869_P01 |  |
|  |  |  |  | GRMZM2G140813_P01 |  |
|  |  |  |  | GRMZM2G150823_P01 |  |
|  |  |  |  | GRMZM2G153556_P01 |  |
|  |  |  |  | GRMZM2G164428_P01 |  |
|  |  |  |  | GRMZM2G165113_P01 |  |
|  |  |  |  | GRMZM2G166354_P01 |  |
|  |  |  |  | GRMZM2G169973_P01 |  |
|  |  |  |  | GRMZM2G178424_P01 |  |

(a, b, c, d, e, f) Gene model of OFP family genes in physcomitrella patens, selaginella, sorghum, arabidopsis, maize and rice, respectively.
