# Supplementary material for: Expression Pattern and Subcellular Localization of the Ovate Protein Family in Rice
Source: PLoS One. 2015 Mar 11;10(3):e0118966. doi: 10.1371/journal.pone.0118966 (PMC4356581; doi:10.1371/journal.pone.0118966)
Supplement: S2 Table — (a, b, c, d) Gene locus of OFP family genes in populus trichocarpa, prunus persica, solanum lycopersicum and cucumis melo, respectively. (DOC) [file pone.0118966.s006.doc]

**Table S2.** Basic information about OFP family genes.

| **Populus trichocarpa (Gene locus)^a^** | **Prunus persica**  **(Gene locus)^b^** | **Solanum lycopersicum**  **(Gene locus)^c^** | **Cucumis melo**  **(Gene locus)^d^** |
| --- | --- | --- | --- |
| Potri.001G180400 | ppa006439m.g | Solyc01g007800 | MELO3C025581P1 |
| Potri.006G158800 | ppa006822m.g | Solyc01g007810 | MELO3C024574P1 |
| Potri.004G200500 | ppa010050m.g | Solyc02g085500 | MELO3C009515P1 |
| Potri.002G051200 | ppa014570m.g | Solyc02g072030 | MELO3C017554P1 |
| Potri.004G062100 | ppa016550m.g | Solyc02g085510 | MELO3C007193P1 |
| Potri.009G161700 | ppa016655m.g | Solyc03g034100 | MELO3C010932P1 |
| Potri.006G107700 | ppa018010m.g | Solyc03g120190 | MELO3C024573P1 |
| Potri.008G153500 | ppa020412m.g | Solyc03g120790 | MELO3C025343P1 |
| Potri.010G087200 | ppa021735m.g | Solyc04g080210 | MELO3C012340P1 |
| Potri.010G241500 | ppa022029m.g | Solyc05g055220 | MELO3C009113P1 |
| Potri.013G155200 | ppa022134m.g | Solyc06g073040 | MELO3C007422P1 |
| Potri.014G181300 | ppa022288m.g | Solyc06g074020 | MELO3C009514P1 |
| Potri.019G128500 | ppa022590m.g | Solyc06g082450 | MELO3C025206P1 |
| Potri.015G004300 | ppa025830m.g | Solyc06g082460 | MELO3C004557P1 |
| Potri.004G003700 | ppa026602m.g | Solyc07g055240 | MELO3C015818P1 |
| Potri.005G125200 | ppa010074m.g | Solyc08g068170 | MELO3C006531P1 |
| Potri.005G211300 |  | Solyc09g018200 | MELO3C019910P1 |
| Potri.006G205400 |  | Solyc09g065350 | MELO3C024232P1 |
| Potri.006G205500 |  | Solyc09g082080 |  |
| Potri.016G072900 |  | Solyc10g076180 |  |
| Potri.007G028000 |  | Solyc10g082050 |  |
| Potri.008G017500 |  | Solyc10g082060 |  |
| Potri.009G161600 |  | Solyc10g083070 |  |
| Potri.016G072800 |  | Solyc10g083080 |  |
| Potri.016G134200 |  | Solyc10g083090 |  |
| Potri.018G080800 |  | Solyc10g083100 |  |
|  |  | Solyc11g006670 |  |
|  |  | Solyc11g068780 |  |
|  |  | SL1.00sc02618_4.1.1 |  |
|  |  | SL1.00sc03540_201.1.1 |  |
|  |  |  |  |
|  |  |  |  |
|  |  |  |  |
|  |  |  |  |
|  |  |  |  |
|  |  |  |  |
|  |  |  |  |
|  |  |  |  |
|  |  |  |  |
|  |  |  |  |
|  |  |  |  |
|  |  |  |  |
|  |  |  |  |
|  |  |  |  |
|  |  |  |  |

(a, b, c, d) Gene locus of OFP family genes in populus trichocarpa, prunus persica, solanum lycopersicum and cucumis melo, respectively.
