# Supplementary material for: Expression Pattern and Subcellular Localization of the Ovate Protein Family in Rice
Source: PLoS One. 2015 Mar 11;10(3):e0118966. doi: 10.1371/journal.pone.0118966 (PMC4356581; doi:10.1371/journal.pone.0118966)
Supplement: S3 Table — (DOC) [file pone.0118966.s007.doc]

***Table S3.*** *Primers for real-time quantitative PCR of OsOFPs.*

| Gene | Forward primer | Reverse primer |
| --- | --- | --- |
| *OsOFP01* | CATTCTTCTCCTCCTCAC | GCTTGTTCCTCTTCTTCT |
| *OsOFP02* | CATCATCATCAAGGTCAGA | TGTTGTTGTCGTCTTCAT |
| *OsOFP03* | ATCCTAGTGCGATCTACTT | GTCGGCATTGGTCTTGTC |
| *OsOFP04* | GACGACGAGTACGACGAG | TTGTAGAAGTCCGACGAGTC |
| *OsOFP05* | GCTTAGAAGTAGTAGACACAACCA | CTCTTGCTGCTCGTGCTT |
| *OsOFP06* | GACAAGTTCGTCGGCTACA | GACCGTCGGCTTCTTGAATT |
| *OsOFP07* | CCATTGCCTCCATCTTCCTCG | AGGCTGTCGGAGCAGTCGTG |
| *OsOFP08* | CAGATGGTGGTGGAGAAG | GTTGAGGGAGAGGAACTG |
| *OsOFP09* | CCGCTTCCTCACCCTCAACG | TGGTGGACGAGGGAGGCAGTA |
| *OsOFP10* | CAGATGATCGTGGAGAAC | GAGTTGAGCGAGAGGAAG |
| *OsOFP11* | TCCTCCTCGCTCCGGTGCAA | CGCCGACGAGTCGACGATGG |
| *OsOFP12* | TGCAGCGACCGTGTCAAAC | GACACGAACCCAAAAAAAGAAAT |
| *OsOFP13* | AGCCTCCTCCTCCTCCTC | TTGCTCTTCTTCTTCCTCCT |
| *OsOFP14* | TCGTCCTGCATTGCTGAC | TCTCCTTCTCCTTCTCCTC |
| *OsOFP15* | CGAGCGGCAGGTTCAGC | CCACCGCCACCAGCACG |
| *OsOFP16* | CGATGCCAAGTTCTCTTC | GCTTCATCATCTTCTCTGT |
| *OsOFP17* | CAGGAGGAGGAGGAAGAC | GTGGTCGTCGAGGAAGAA |
| *OsOFP18* | CTCTAGAAGCCTCGTCTC | CTTGCTCTTGTGGGGAAG |
| *OsOFP19* | GAACGAGCCAAGCAGATG | GGATTCTTTGGTGGAGAGG |
| *OsOFP20* | GATGGTGGTGGAGAAGGA | GAGTTGAGGGTCAGGAAC |
| *OsOFP21* | CCTCTTCTGTTCCGACTC | GTGTTTGTTTGTGTGTATGTG |
| *OsOFP22* | ACCGTTCAGCGAGACAAG | ATAAGTAGCGGCGGGAGA |
| *OsOFP23* | AGCGGCAGGCGGTCGTTC | GCACCACCGCCAGCCTCTC |
| *OsOFP24* | GCTTTTCCACCGCAACATCT | CGAGGTTGCACCCCACTT |
| *OsOFP25* | ATAGGAAGAAGAAGAAGAC | TAGTAGTAGTGGATGTGT |
| *OsOFP26* | CCTTTCTTGACGACCTT | GGGTGCTCGGCGGAATC |
| *OsOFP27* | GCTCAAGTTTTTCGTCATC | TACGAAAATACGAACTGAAAT |
| *OsOFP28* | CGAGGAAGAAGAAGAAGA | AATGTACTTGGTGCTAAG |
| *OsOFP29* | TGATAGAATCTCTCCAACG | GTACCAGCCAACTTTACTTAA |
| *OsOFP30* | GGATGAGGAAGCTGGTTG | TTATGGCCGAGACCTCTT |
| *OsOFP31* | ATTGCTAATAATCTCTCTGTT | TAAAAATCATCAAGAGCAC |
| *OsActin* | TTCCAGCCTTCCTTCATA | AACGATGTTGCCATATAGAT |
