# Supplementary material for: Expression Pattern and Subcellular Localization of the Ovate Protein Family in Rice
Source: PLoS One. 2015 Mar 11;10(3):e0118966. doi: 10.1371/journal.pone.0118966 (PMC4356581; doi:10.1371/journal.pone.0118966)
Supplement: S4 Table — (DOC) [file pone.0118966.s008.doc]

**Table S4.** Primers for systemic subcellular localization assays of OsOFPs.

| Gene | Forward primer | Reverse primer |
| --- | --- | --- |
| *OsOFP01* | CAAAAAAGCAGGCTTCATGGCGATGGATCATCGTGG | CAAGAAAGCTGGGTCGCCGCCATGGAGGACGAC |
| *OsOFP02* | CAAAAAAGCAGGCTTCATGCAAGAAGATAACATCAGTA | CAAGAAAGCTGGGTCATAGTGTTTCCTACTCCGG |
| *OsOFP03* | CAAAAAAGCAGGCTTCATGCACACGCCTTTCGTGGACA | CAAGAAAGCTGGGTCCTCCTCGCCGAAGACGGCCT |
| *OsOFP04* | CAAAAAAGCAGGCTTCATGGACGGCGGCGGCT | CAAGAAAGCTGGGTCGGGGAAAAGCGTCTCCCGC |
| *OsOFP05* | CAAAAAAGCAGGCTTCATGAAGTGGGGGCTTAGAAGTAGTA | CAAGAAAGCTGGGTCGAGCTCGAACCAGACCTGCC |
| *OsOFP06* | CAAAAAAGCAGGCTTCATGGGGAGGCACAAGTTCAGG | CAAGAAAGCTGGGTCCATCTTGATGTCCGCGAGGTTG |
| *OsOFP07* | CAAAAAAGCAGGCTTCATGGTCATCGGAAGGCTGGC | CAAGAAAGCTGGGTCCACGGCGAGTTTTGCCTGCT |
| *OsOFP08* | CAAAAAAGCAGGCTTCATGTCGGGCAGGTCGTCAAG | CAAGAAAGCTGGGTCGAACTGGCAGGGGGACGG |
| *OsOFP09* | CAAAAAAGCAGGCTTCATGATGAGTCCTGGCGTTTCGGC | CAAGAAAGCTGGGTCCACGCGCCATGCCCGTG |
| *OsOFP10* | CAAAAAAGCAGGCTTCATGGCCGATCAGGGCCTAC | CAAGAAAGCTGGGTCGTTGGTAGTGGCGCTGTACGG |
| *OsOFP11* | CAAAAAAGCAGGCTTCATGCTGGGCTGCTTCTCC | CAAGAAAGCTGGGTCATGTGACTCGTGCAGGTGG |
| *OsOFP12* | CAAAAAAGCAGGCTTCATGGCCAAGCGTCTCTTCCA | CAAGAAAGCTGGGTCCCGACGGTTTGACACGGTC |
| *OsOFP13* | CAAAAAAGCAGGCTTCATGCCAGCCTCCTCCTCCTCC | CAAGAAAGCTGGGTCCTGGCCGGGAAGAAGCCTCTG |
| *OsOFP14* | CAAAAAAGCAGGCTTCATGCCTCCCCTGAACCCTC | CAAGAAAGCTGGGTCGCTCTCCTCCTCCATGGTCG |
| *OsOFP15* | CAAAAAAGCAGGCTTCATGGTCCAGGCGAGGCTGCAGT | CAAGAAAGCTGGGTCGCGCTTGCGGGAGAAGAGGC |
| *OsOFP16* | CAAAAAAGCAGGCTTCATGAGCCCCAGCGCCGC | CAAGAAAGCTGGGTCCAGGCGCCAAGCCGGTAGAG |
| *OsOFP17* | CAAAAAAGCAGGCTTCATGGTTCGGAAGCTTGTTGC | CAAGAAAGCTGGGTCGTTAATCTGGCTGCCTTTCTGG |
| *OsOFP18* | CAAAAAAGCAGGCTTCATGGACGACGGAGCCAAGAGC | CAAGAAAGCTGGGTCGCCGCCGCGGAGAACGA |
| *OsOFP19* | CAAAAAAGCAGGCTTCATGAGCAGCCATGAGAGGTTC | CAAGAAAGCTGGGTCCAACCCAACGCAACAATCAG |
| *OsOFP20* | CAAAAAAGCAGGCTTCATGGCGCCATTGATGTCAG | CAAGAAAGCTGGGTCGAGAGCTTGGCACGGGGA |
| *OsOFP21* | CAAAAAAGCAGGCTTCATGCATGTCCTCTTCTGTTCC | CAAGAAAGCTGGGTCCCTTAGCCTGAAAGAGATATGG |
| *OsOFP22* | CAAAAAAGCAGGCTTCATGTTGTCCAGCGAACCAGG | CAAGAAAGCTGGGTCTGGCATGACGCCACAGG |
| *OsOFP23* | CAAAAAAGCAGGCTTCATGGGATGGGGGATCAG | CAAGAAAGCTGGGTCGCAATGGCGAGCTCGTC |
| *OsOFP24* | CAAAAAAGCAGGCTTCATGTCAGATTCTTGCAAGATG | CAAGAAAGCTGGGTCCTTGATCGGGCTAGGTG |
| *OsOFP25* | CAAAAAAGCAGGCTTCATGGTGAATAGGAAGAAGAAGAAGA | CAAGAAAGCTGGGTCGTAATGTCGTAGCTGATGTTCTTCT |
| *OsOFP26* | CAAAAAAGCAGGCTTCATGGCGGGGTGCTTGC | CAAGAAAGCTGGGTCTGCCTTCCTCCTTGCGTC |
| *OsOFP27* | CAAAAAAGCAGGCTTCATGAAGGTGATGACACTGCGGC | CAAGAAAGCTGGGTCGGCGGCGTTCCACGAGTC |
| *OsOFP28* | CAAAAAAGCAGGCTTCATGGCCAAGAAGAAGGGCTT | CAAGAAAGCTGGGTCAGAATTAGCAAATGTACTTGGTGCT |
| *OsOFP29* | CAAAAAAGCAGGCTTCATGTCCACGTCCATGGCG | CAAGAAAGCTGGGTCGCTGCGCCGGTGATGG |
| *OsOFP30* | CAAAAAAGCAGGCTTCATGGGCAAGAAGGGTGGC | CAAGAAAGCTGGGTCGCTTATGGCCGAGACCTCTTC |
| *OsOFP31* | CAAAAAAGCAGGCTTCATGTCAACGGCGGCGAG | CAAGAAAGCTGGGTCGTAGTAGTGGTAGTGGTACGCCGC |
